# The dosimetric impact of deep learning-based auto-segmentation of organs at risk on nasopharyngeal and rectal cancer

**DOI:** 10.1186/s13014-021-01837-y

**Published:** 2021-06-23

**Authors:** Hongbo Guo, Jiazhou Wang, Xiang Xia, Yang Zhong, Jiayuan Peng, Zhen Zhang, Weigang Hu

**Affiliations:** 1 Department of Radiation Oncology, Fudan University Shanghai Cancer Center, Shanghai, 200032 China; 2 Department of Oncology, Shanghai Medical College, Fudan University, Shanghai, 200032 China; 3 Shanghai Key Laboratory of Radiation Oncology, Shanghai, 200032 China

**Keywords:** Treatment planning, Dosimetric, Deep learning, Auto-segmentation

## Abstract

**Purpose:**

To investigate the dosimetric impact of deep learning-based auto-segmentation of organs at risk (OARs) on nasopharyngeal and rectal cancer.

**Methods and materials:**

Twenty patients, including ten nasopharyngeal carcinoma (NPC) patients and ten rectal cancer patients, who received radiotherapy in our department were enrolled in this study. Two deep learning-based auto-segmentation systems, including an in-house developed system (FD) and a commercial product (UIH), were used to generate two auto-segmented OARs sets (OAR_FD and OAR_UIH). Treatment plans based on auto-segmented OARs and following our clinical requirements were generated for each patient on each OARs sets (Plan_FD and Plan_UIH). Geometric metrics (Hausdorff distance (HD), mean distance to agreement (MDA), the Dice similarity coefficient (DICE) and the Jaccard index) were calculated for geometric evaluation. The dosimetric impact was evaluated by comparing Plan_FD and Plan_UIH to original clinically approved plans (Plan_Manual) with dose-volume metrics and 3D gamma analysis. Spearman’s correlation analysis was performed to investigate the correlation between dosimetric difference and geometric metrics.

**Results:**

FD and UIH could provide similar geometric performance in parotids, temporal lobes, lens, and eyes (DICE, *p* > 0.05). OAR_FD had better geometric performance in the optic nerves, oral cavity, larynx, and femoral heads (DICE, *p* < 0.05). OAR_UIH had better geometric performance in the bladder (DICE, *p* < 0.05). In dosimetric analysis, both Plan_FD and Plan_UIH had nonsignificant dosimetric differences compared to Plan_Manual for most PTV and OARs dose-volume metrics. The only significant dosimetric difference was the max dose of the left temporal lobe for Plan_FD vs. Plan_Manual (*p* = 0.05). Only one significant correlation was found between the mean dose of the femoral head and its HD index (R = 0.4, *p* = 0.01), there is no OARs showed strong correlation between its dosimetric difference and all of four geometric metrics.

**Conclusions:**

Deep learning-based OARs auto-segmentation for NPC and rectal cancer has a nonsignificant impact on most PTV and OARs dose-volume metrics. Correlations between the auto-segmentation geometric metric and dosimetric difference were not observed for most OARs.

**Supplementary Information:**

The online version contains supplementary material available at 10.1186/s13014-021-01837-y.

## Introduction

Organs at risk (OARs) delineation is a critical task in radiotherapy. It affects many aspects of treatment planning, which can further affect the probability of local tumor control and normal tissue complications [1–4]. However, manual OARs delineation is time-consuming and tedious work. This fact is especially true for cancers with complex anatomy, such as nasopharyngeal carcinoma (NPC).

Auto-segmentation can reduce the work intensity of oncologists and improve work efficiency [5–10]. Recently, deep learning-based auto-segmentation has become a mainstream assistance segmentation technique provided by many software vendors [7, 11–13]. The latest relevant studies have shown promising results for these systems, improving consistency among oncologists and shortening the delineation time [14–16].

As an emerging technique, sufficient clinical application assessment is required. Although many studies have evaluated the performance of auto-segmentation in terms of geometric metrics [7, 14, 15, 17–19], few studies have focused on dosimetric impact [11, 20]. Because the OARs delineation directly affects the plan optimization and local dose distribution, and then affects the plan evaluation and the normal tissue complication probability. Therefore, dosimetry evaluation has important clinical significance, only geometric metric evaluation is not sufficient for clinical application.

There are many approaches for dosimetry evaluation of OARs auto-segmentation. Van Dijk et al. [11] compared the dosimetric difference between auto-segmented and manually delineated OARs with a clinically approved treatment plan. The results proved that more accurate auto-segmentation translated into smaller dosimetric differences compared to the manual contours. Kaderka et al. [20] used an atlas-based method for cardiac substructure segmentation and proved that the quality of auto-segmented contours cannot be determined by geometric metrics only, and geometrical measures did not predict the accuracy of dosimetric parameters. However, both two studies used clinically approved treatment plans based on manual delineation and assessed on auto-segmented contours.

The future goal of OARs auto-segmentation is to be applied to clinical plan optimization and evaluation with little or no manual modification. The OARs delineation will directly affect the plan optimization and local dose distribution. Re-optimizing the plan based on auto-segmented OARs is more in line with the actual clinical situation, so we think it may be the most reasonable approach. However, the existing researches have not evaluated the feasibility of applying the auto-segmented OARs to plan optimization.

We believe that the feasibility evaluation of applying the auto-segmented OARs to plan optimization has important clinical significance, because it is the basis of the whole process automation of treatment planning (including automatic delineation, automatic planning, plan evaluation, etc.), and this paper has conducted a preliminary exploration on this. In this study, we reoptimized the treatment plan based on auto-segmented contours and then used manual contours to evaluate the dosimetric differences between the reoptimized plans and the original clinical treatment plans.

To further assess the dosimetric impact of deep learning-based auto-segmentation, we have designed a dosimetric comparison study. Two sites, including the nasopharynx and rectum, and two deep learning-based auto-segmentation systems, including a commercial tool from United Imaging Healthcare (UIH, Shanghai, China) and an in-house auto-segmentation tool developed by our institution, were investigated. To evaluate the application of deep learning-based auto-segmentation in clinical situations, the whole planning process was following our clinical routine requirement. Meanwhile, the correlation between geometric metric and dosimetric difference was investigated.

## Methods

**Fig. 1:**
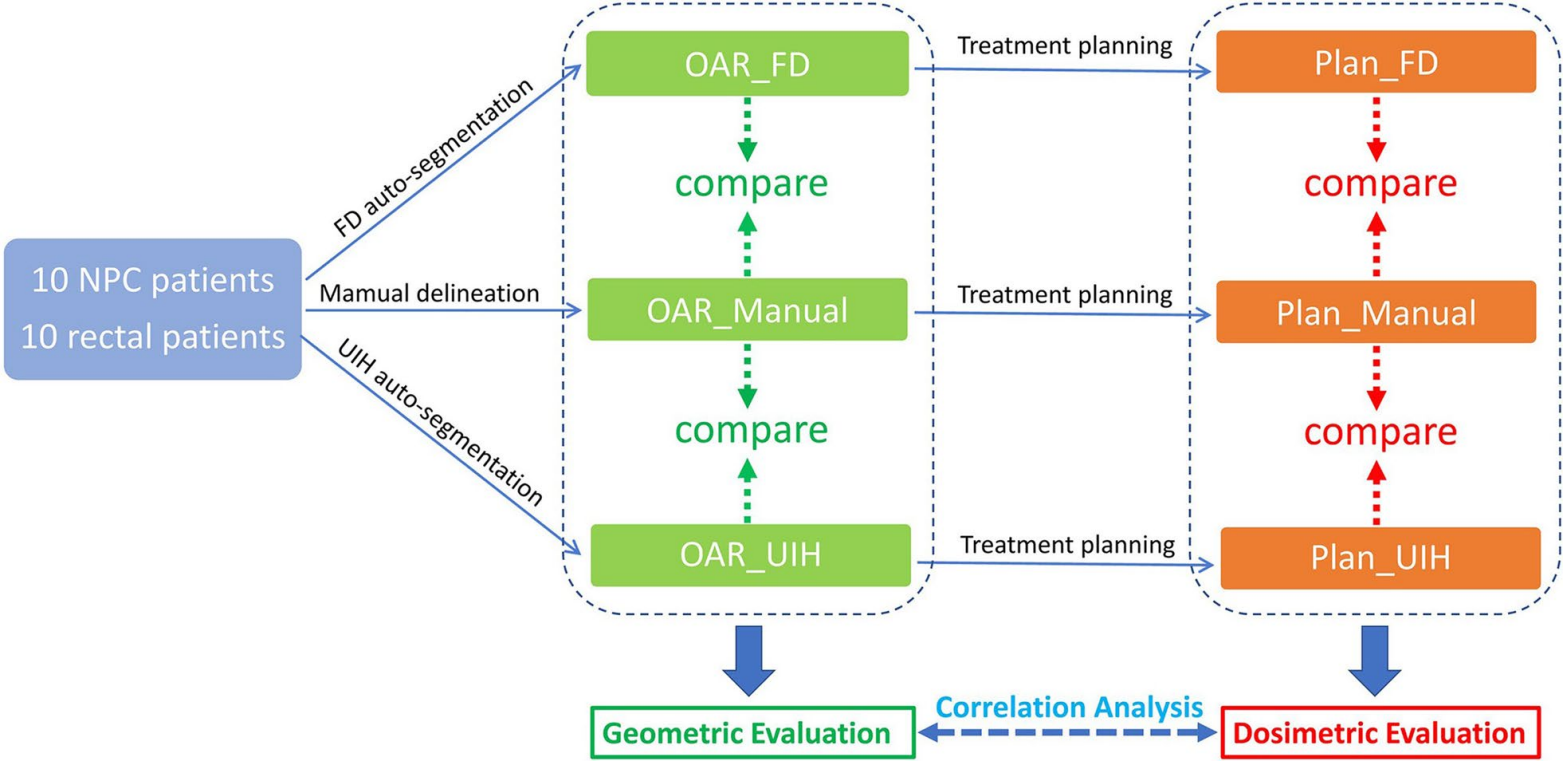
The workflow of this study

A schematic workflow of this study is presented in Fig. 1. After auto-segmentation, the assessment was divided into three parts. First, the accuracy of auto-segmentation was evaluated based on geometric metrics. Second, we reoptimized the plan based on the auto-segmented OARs and compared it with the original treatment plan to evaluate the dosimetric differences. Third, we explored the correlation between the geometric metrics and dosimetric differences.

### Patients and treatment protocol

Two sites, including the nasopharynx and rectum, were investigated. Ten patients for each site who received radiotherapy at Fudan University Shanghai Cancer Center between 2017 and 2019 were randomly selected from our database and enrolled in this study. The details of the patient characteristics are shown in Additional file 1: Supplement A, Table S1. For NPC patients, the prescription was 70.4 Gy in 32 fractions for T3-T4 stage patients and 66 Gy in 30 fractions for T1-T2 stage patients. For rectal cancer, all of the patients received 50 Gy in 25 fractions.

### OARs manual delineation

Manual delineation was performed on the Pinnacle (Pinnacle, v9.10, Philips Corp, Fitchburg, WI, USA) treatment planning system. The targets and OARs are presented in Table 1. These contours were delineated by radiation oncologists with more than 5 years of experience in radiation oncology and revised and approved by senior radiation oncologists. All of the manually delineated OARs were used for patient treatment.

**Table 1:** The target and the OARs constraint functions and dosimetric evaluation metrics

Site	Target/OARs	Prescription	Constraints or objectives	Dosimetric evaluation
NPC	PTV70.4	70.4 Gy/32 F	D_95_ > Prescription, D_2_ < 110% Prescription, uniform dose = Prescription	V_100_, D_95_, D_2_
	PTV66	66 Gy/32 F		
	PTV60	60 Gy/32 F		
	PTV54	54 Gy/32 F		
	Eyes	/	Max Dose < 50 Gy	Max Dose
	Lens	/	Max Dose < 25 Gy	Max Dose
	Brainstem	/	Max Dose < 54 Gy	Max Dose
	Temporal lobes	/	Max Dose < 65 Gy, V_60_ < 1%	Max Dose
	Spinal cord	/	Max Dose < 45 Gy	Max Dose
	Optic nerves	/	Max Dose < 54 Gy	Max Dose
	Larynx	/	Mean Dose < 45 Gy	Mean Dose
	Parotid	/	Mean Dose < 26 Gy	Mean Dose, V_30_
	Oral cavity	/	Mean Dose < 40 Gy	Mean Dose
	Temporomandibular joints	/	Max Dose < 70 Gy	Not evaluated
	Chiasm	/	Max Dose < 54 Gy	Not evaluated
Rectum	PTV	50 Gy/25 F	D_95_ > Prescription, D_2_ < 110% Prescription, uniform dose = Prescription	V_100_, D_95_, D_2_
	Femoral heads	/	V_40_ < 40%, Max Dose < 50 Gy	Mean Dose
	Bladder	/	V_40_ < 50%	Mean Dose, V_40_

### Deep learning-based auto-segmentation

Two deep learning-based auto-segmentation systems were used in this study. FD is an in-house developed deep learning-based auto-segmentation system, the details of the network and model training have been presented in our recent studies [21–24]. Briefly, we used approximately 200 NPC and 200 rectal cancer cases from our institution as the training dataset. The delineation of the training dataset came from clinical routine without modification for this task. The network was a modified 2D U-Net. It was used in 2018 for OARs auto-segmentation clinical testing on NPC and rectal cancer. The OARs segmented by this system were marked as OAR_FD.

UIH is a commercial treatment planning system developed by UIH Corporation [25, 26]. It uses a two-phase 3D U-Net for OARs location and segmentation. The training data did not come from our institution. We used UIH from 2019 for clinical testing. This system provided NPC and rectal cancer OARs auto-segmentation, which was used in this study. The OARs segmented by this system were marked as OAR_UIH.

### Treatment planning

Pinnacle (Pinnacle, v9.10, Philips Corp, Fitchburg, WI, USA) and Varian Trilogy Linac (Varian, Polo Alto, CA, USA) with 120 multileaf collimator were used for treatment planning for all plans. All of the treatment planning processes were the same as our clinical routine for consistency.

The NPC clinical treatment plans used the 9-field static intensity modulated radiotherapy (sIMRT) technique, and the gantry angles were 0°, 45°, 85°, 120°, 160°, 200°, 245°, 275°, and 315°. The field could be split based on field width. The maximum number of segmented subfields was set to 55. The rectal cancer clinical treatment plans adopted 7 fields of the sIMRT technique. The beam angles were chosen based on clinical experience. Here, we mainly considered having the bladder and femoral heads receive less radiation exposure. The maximum number of segmented subfields was set to 35. For all of the plans, the minimum subfield area was set to 10 cm^2^, and the minimum subfield monitoring unit was set to 10 MU. The dose calculation grid was set to 3 mm.

The prescription was normalized to the mean dose of PTV as in our clinical routine. For NPC, we prescribe 220 cGy per fraction to 97% of the PTV70.4 mean dose for 32 fractions or 220 cGy per fraction to 97% of the PTV66 mean dose for 30 fractions. For rectal cancer, we prescribe 200 cGy per fraction to 96% of PTV mean dose for 25 fractions. In this setting, the D_95_ of PTV was close to the prescription dose. All of the treatment plans were completed by medical physicists with more than 3 years of experience.

Each patient had three plans: Plan_Manual, Plan_FD and Plan_UIH. Plan_Manual was a clinically approved plan that was used for patient treatment. Plan_FD and Plan_UIH were reoptimized based on manually delineated PTVs and auto-segmented OARs. For OARs that were not generated by the auto-segmentation system (temporomandibular joints and chiasm), we used manually delineated OARs to replace them. The beam angles and initial optimization parameters for the reoptimized plans (Plan_FD and Plan_UIH) were consistent with Plan_Manual. The physicist could adjust the optimization objective function based on his or her experience and judgment, the same as the routine clinical treatment planning process.

### Geometric evaluation

Manual delineated contours were used as references. The performance of auto-segmentation was evaluated by the following four geometric metrics: Hausdorff distance (HD), mean distance to agreement (MDA), Dice similarity coefficient (DICE), and Jaccard index [27–29]. HD and MDA were used to quantify the maximum and mean 3D distances between contours A and B, respectively. DICE and the Jaccard index were measures of the overlap between contours A and B. The definitions are as follows:

$${\text{HD~}}\left( {{\text{A}},{\text{~B}}} \right) = max\left\{ {H~\left( {A,~B} \right),~~~H\left( {B,~A} \right)} \right\}$$

$$H\left( {A,~B} \right)~ = ~{}_{{a\epsilon A}}^{{max}} \left\{ {{}_{{b \epsilon B}}^{{min}} \left\{ {d~\left( {a,b} \right)} \right\}} \right\}$$

where *d* (a, b) represents the 3D Hausdorff distance between point a from contour A and point b from contour B.

$${\text{MDA~}}\left( {{\text{A}},{\text{~B}}} \right)~ = ~\frac{{h~\left( {A,~B} \right)~ + ~h~\left( {B,~A} \right)}}{2}$$

$$h~\left( {A,~B} \right)~ = ~{}_{{a\epsilon A}}^{{mean}} \left\{ {{}_{{b \epsilon B}}^{{min}} \left\{ {d~\left( {a,b} \right)} \right\}} \right\}$$

$${\text{DICE}}~ = ~2~*~\frac{{\left| {{\text{A}} \cap {\text{B}}} \right|}}{{\left| {\text{A}} \right| + \left| {\text{B}} \right|}}$$

$${\text{Jaccard}}~ = ~~\frac{{\left| {{\text{A}} \cap {\text{B}}} \right|}}{{\left| {{\text{A}} \cup {\text{B}}} \right|}}$$

For a perfect overlap, the values of HD and MDA are 0, and the values of DICE and Jaccard are 1. For an imperfect overlap, the values of HD and MDA are large, and the values of DICE and Jaccard are close to 0.

### Dosimetric evaluation

**Fig. 2:**
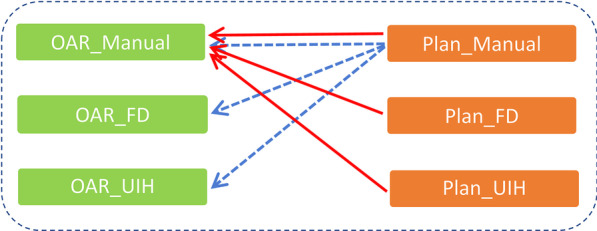
Different dosimetric evaluation methods between this study and others

Plan_Manual was clinically approved treatment plan, Plan_FD and Plan_UIH were reoptimized plans based on manually delineated PTVs and auto-segmented OARs. In the dosimetric evaluation, we used manually delineated OARs to compare dose-volume metrics between Plan_FD, Plan_UIH and Plan_Manual. As Fig. 2 shows, the red solid line represents the method we used in this study, the blue dash line represents the traditional evaluation method. For serial organs, we mainly focused on D_max_. For parallel organs, we mainly focused on D_mean_, V_30_ or V_40_ (Table 1). The dose-volume metrics of PTVs and manually delineated OARs were extracted from Plan_FD, Plan_UIH and Plan_Manual.

A 3D gamma analysis was performed with 3% and 3 mm for whole-body and PTV dose distribution comparison. The homogeneity index (HI) and conformity index (CI) for PTV were further calculated using the following formulas:

$${\text{HI}} = \frac{{{\text{D}}_{2} - {\text{D}}_{{98}} }}{{{\text{D}}_{{\text{p}}} }}$$

$${\text{CI}} = \frac{{{\text{V}}_{{\text{R}}} {\text{*V}}_{{\text{R}}} }}{{{\text{V}}_{{\text{P}}} {\text{*V}}_{{{\text{dose}}}} }}$$

where D_P_ is the prescription dose, V_P_ and V_dose_ are the volume of PTV and the prescription dose region, respectively, and V_R_ is the intersection volume of V_P_ and V_dose_.

### Correlation between the geometric metric and dosimetric metric

The correlation between the geometric metric and the ∆Dose was analyzed by Spearman’s correlation test. The ∆Dose is the dose-volume metrics difference between reoptimized plans (including Plan_FD and Plan_UIH) and Plan_Manual. Please note that the volume-metrics difference is also denoted by ΔDose.

### Statistical analysis

R software (v4.0) was used for statistical analysis. For a value comparison, the Shapiro–Wilk normality test was performed first. If a normal distribution was found, the paired-sample t test between groups was performed; otherwise, the Wilcoxon’s paired-sample nonparametric signed-rank test was performed. *p* < 0.05 indicates that the difference is statistically significant. The correlations between geometric metrics and dose-volume metrics difference were evaluated with Spearman’s correlation coefficient R.

## Results

### Geometric evaluation

**Fig. 3:**
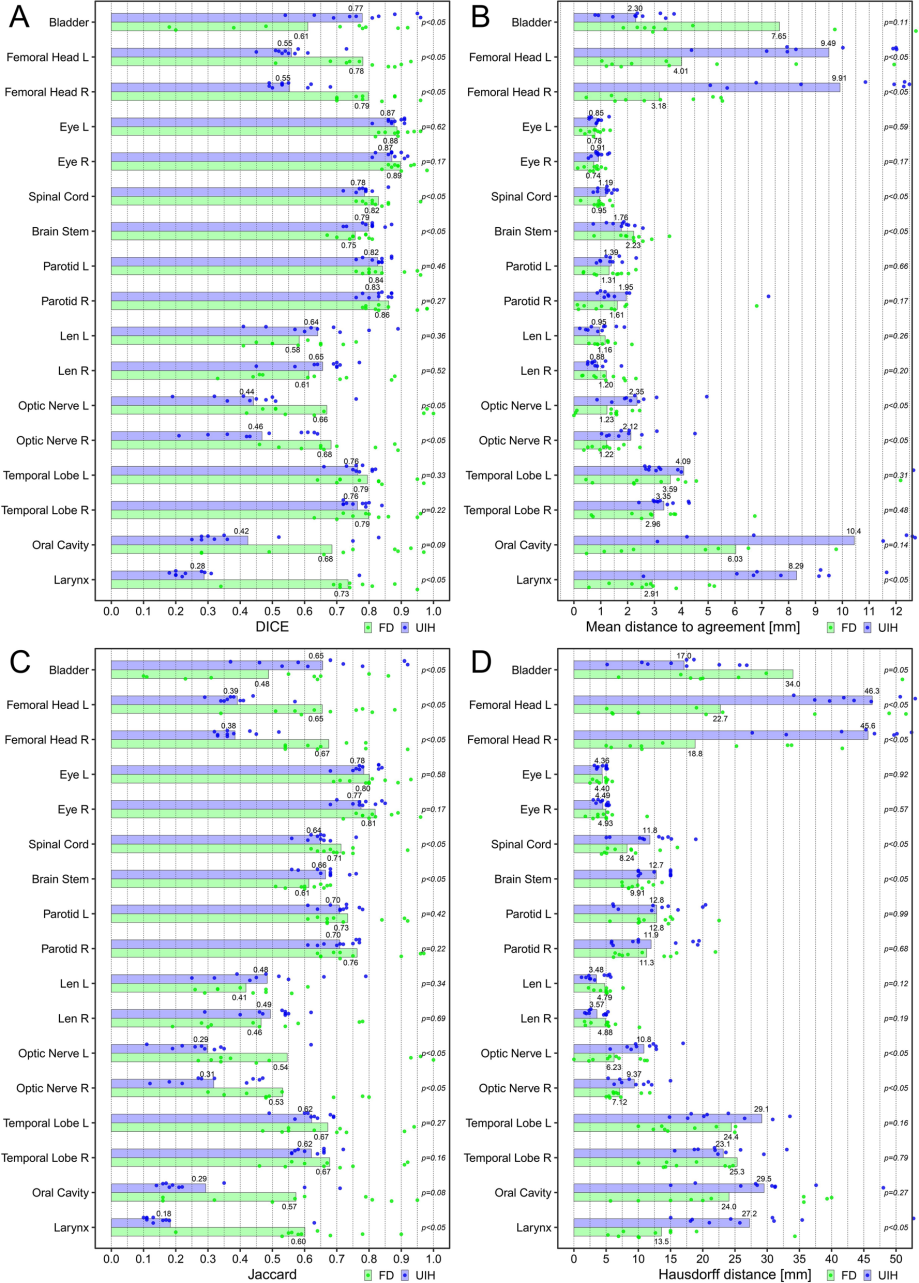
Geometric evaluation results of auto-segmentation. **a** The DICE; **b** The mean distance to agreement (MDA); **c** The Jaccard; **d** The Hausdorff distance (HD)

**Fig. 4:**
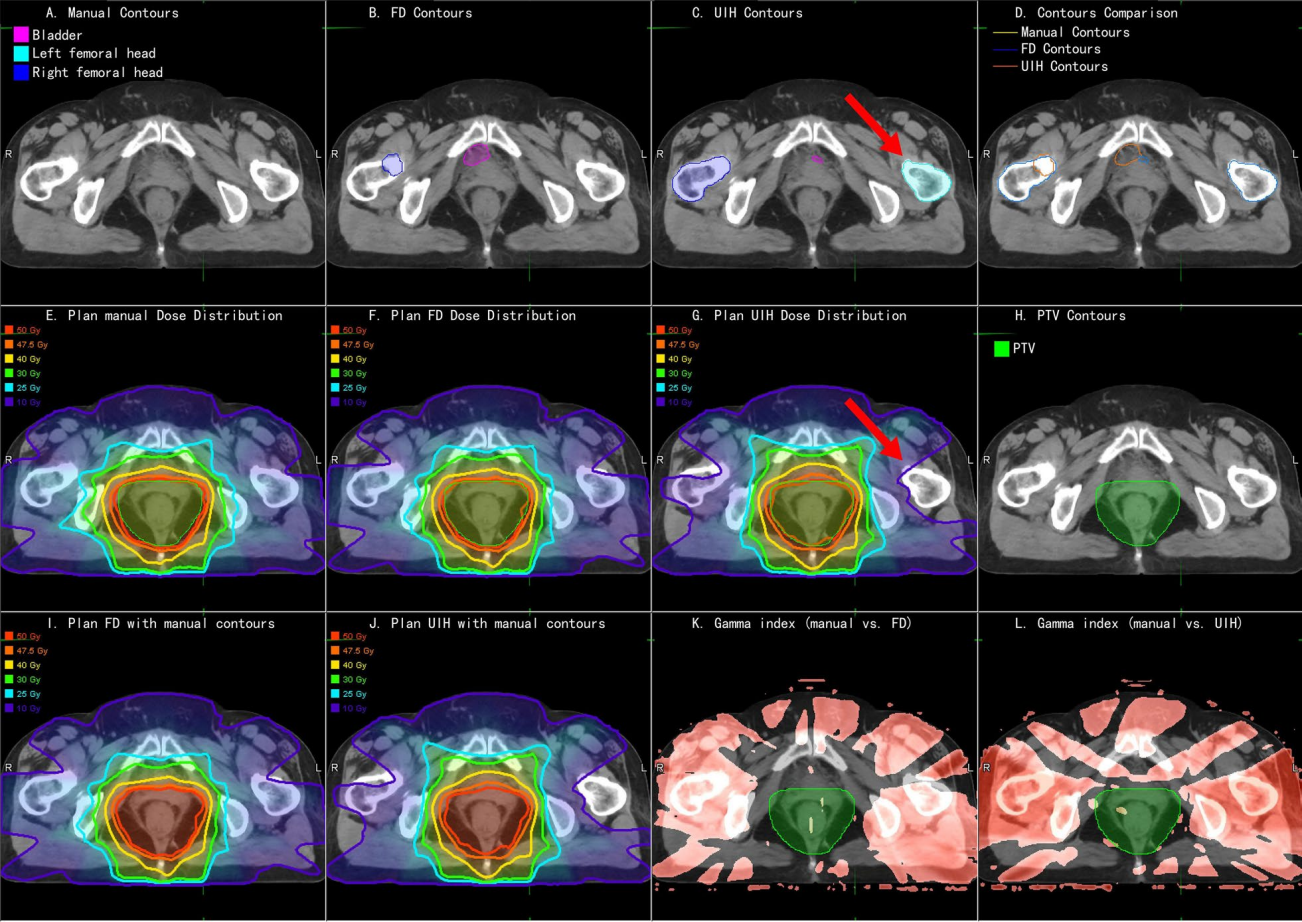
An example of rectal cancer patient. **a** Manual OARs; **b** FD OARs; **c** UIH OARs; **d** Contour comparison; **e** Plan_Manual dose distribution; **f** Plan_FD dose distribution; **g** Plan_UIH dose distribution; **h** PTV contour; **i** Plan_FD with manual OARs; **j** Plan_UIH with manual OARs; **k** 3D Gamma analysis of Plan_FD; red color represents gamma index > 1; **l** 3D Gamma analysis of Plan_UIH, red color represents gamma index > 1

Figure 3 shows the geometric evaluation results of auto-segmentation. Both deep learning systems can provide similar results in some OARs, including the parotids, temporal lobes, lens, and eyes (DICE, *p* > 0.05). Here, the *p*-Value indicates the DICE difference between OAR_FD and OAR_UIH. For the brainstem and spinal cord, although there was a significant difference (*p* < 0.05), the deviation was small (less than 0.05 in DICE), while OAR_FD had better performance in the optic nerves, oral cavity, larynx, and femoral heads. OAR_UIH had better performance in the bladder. Representative rectal cancer and NPC examples of auto-segmentation are illustrated in Fig. 4 and Additional file 1: Supplement B, Fig. S1. More examples are presented in Additional file 1: Supplement D, Figs. S6-S12.

### PTV dosimetry evaluation

Table 2 lists the PTV dosimetric parameters of Plan_Manual, Plan_FD and Plan_UIH. No significant dosimetric differences were found by comparison Plan_FD, Plan_UIH with Plan_Manual.

**Table 2:** Summary of the PTV dosimetry parameters of the reoptimized treatment plans (Plan_FD and Plan_UIH) and the original clinical treatment plans (Plan_Manual). All of the values are reported as the mean ± standard deviation

Site/structure	Dosimetric Indices	Plan	Value	Comparison (paired t test)
Rectal/PTV	V_100_ (%)	Manual	97.41 ± 1.91	–
		FD	96.37 ± 2.98	*p* = 0.35
		UIH	96.49 ± 2.72	*p* = 0.33
	D_95_ (cGy)	Manual	5088 ± 77	–
		FD	5028 ± 66	*p* = 0.09
		UIH	5042 ± 70	*p* = 0.11
	D_2_ (cGy)	Manual	5349 ± 177	–
		FD	5384 ± 167	*p* = 0.08
		UIH	5383 ± 160	*p* = 0.08
	Gamma Pass Rate (3 mm/3%)	FD	97.16 ± 2.43	–
		UIH	97.20 ± 2.34	–
	Conformity Index (CI)	Manual	0.78 ± 0.23	–
		FD	0.78 ± 0.23	*p* = 0.52
		UIH	0.78 ± 0.23	*p* = 0.48
	Homogeneity Index (HI)	Manual	0.10 ± 0.08	–
		FD	0.10 ± 0.06	*p* = 0.64
		UIH	0.10 ± 0.06	*p* = 0.63
NPC/PTV70.4	V_100_ (%)	Manual	93.39 ± 0.94	–
		FD	92.75 ± 2.03	*p* = 0.39
		UIH	92.28 ± 2.00	*p* = 0.12
	D_95_ (cGy)	Manual	6984 ± 25	–
		FD	6978 ± 31	*p* = 0.66
		UIH	6962 ± 32	*p* = 0.16
	D_2_ (cGy)	Manual	7425 ± 270	–
		FD	7445 ± 287	*p* = 0.17
		UIH	7423 ± 276	*p* = 0.92
	Gamma Pass Rate (3 mm/3%)	FD	99.20 ± 1.07	–
		UIH	96.35 ± 2.34	–
	Conformity Index (CI)	Manual	0.72 ± 0.05	–
		FD	0.70 ± 0.04	*p* = 0.23
		UIH	0.70 ± 0.06	*p* = 0.24
	Homogeneity Index (HI)	Manual	0.12 ± 0.05	–
		FD	0.12 ± 0.05	*p* = 0.33
		UIH	0.12 ± 0.04	*p* = 0.58

### OARs dosimetry evaluation

**Fig. 5:**
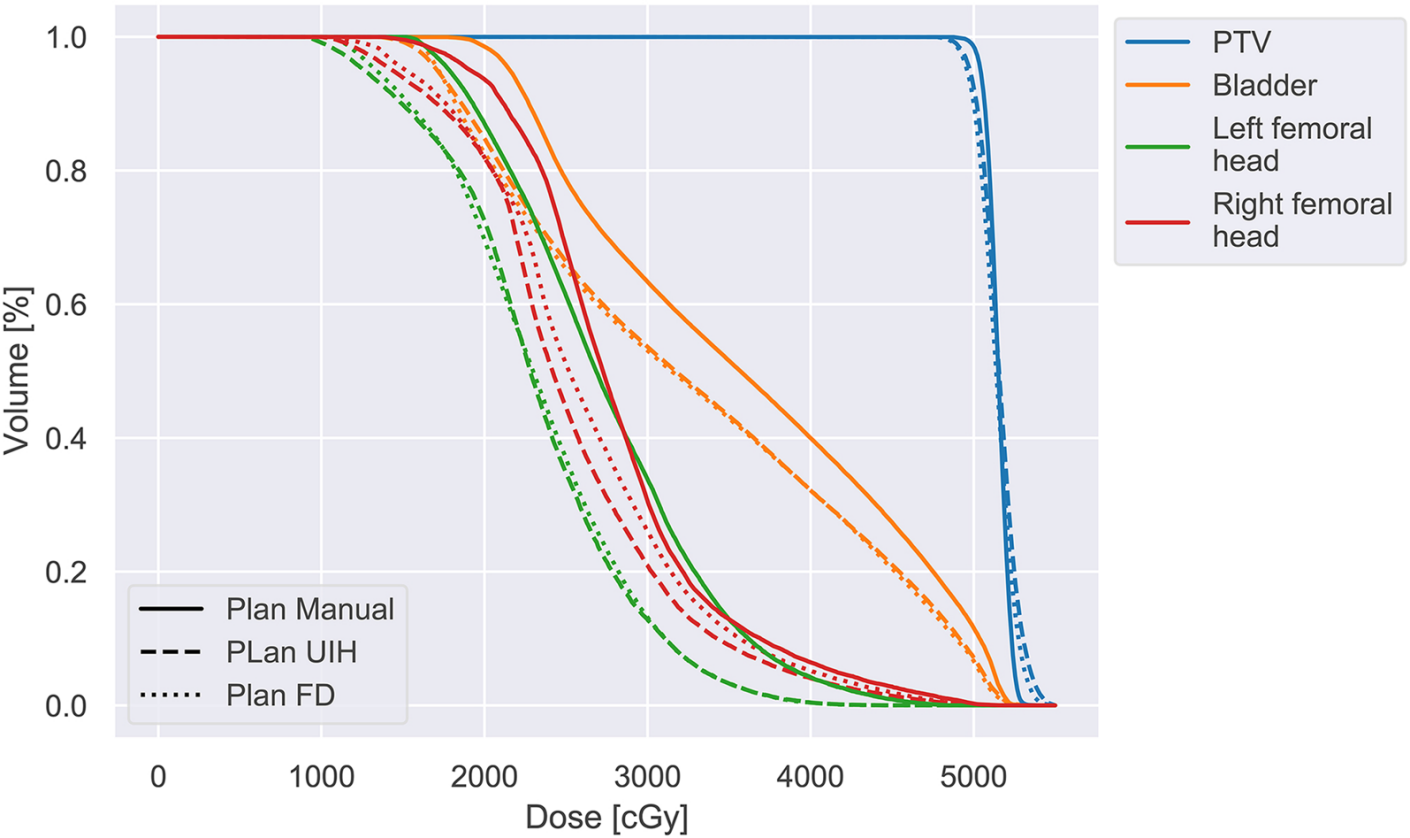
The dose-volume histogram (DVH) of Plan_Manual, Plan_FD and Plan_UIH for an representative rectal cancer cases

Table 3 lists the OARs dosimetry parameters. No significant dosimetric differences were found except for left temporal lobe D_max_ for Plan_FD vs. Plan_Manual (6376 ± 2126 cGy vs. 6444 ± 2156 cGy, *p* = 0.05). Figure 4 and Additional file 1: Supplement B, Fig. S1 present the dose distributions of Plan_Manual, Plan_FD and Plan_UIH for representative rectal cancer and NPC cases. Figure 5 shows an example of dose-volume histogram (DVH) of Plan_Manual, Plan_FD and Plan_UIH for representative rectal cancer cases. If readers are interested in the dose-volume metrics data of Plan_Manual on OAR_FD and OAR_UIH, please refer to Additional file 1: Supplement B, Table S2.

**Table 3:** Summary of the OARs dosimetry parameters of the reoptimized treatment plans (Plan_FD and Plan_UIH) and the original clinical treatment plans (Plan_Manual). All of the values are reported as the mean ± standard deviation

Site	Structure	Dose-volume metrics	Plan	Value	Comparison (paired t test)
Rectal	Bladder	V_40_ (%)	Manual	43.45 ± 26.96	–
			FD	43.73 ± 26.28	*p* = 0.93
			UIH	41.76 ± 26.55	*p* = 0.14
		D_mean_ (cGy)	Manual	3476 ± 1281	–
			FD	3464 ± 1264	*p* = 0.82
			UIH	3427 ± 1262	*p* = 0.25
	Femoral head_L	D_mean_ (cGy)	Manual	2199 ± 773	–
			FD	2160 ± 718	*p* = 0.60
			UIH	2185 ± 824	*p* = 0.90
	Femoral head_R	D_mean_ (cGy)	Manual	2130 ± 755	–
			FD	2085 ± 712	*p* = 0.41
			UIH	2153 ± 819	*p* = 0.79
NPC	Eye_L	D_max_ (cGy)	Manual	2199 ± 1175	–
			FD	2135 ± 1265	*p* = 0.56
			UIH	2153 ± 1168	*p* = 0.71
	Eye_R	D_max_ (cGy)	Manual	2441 ± 1892	–
			FD	2386 ± 1906	*p* = 0.61
			UIH	2463 ± 1906	*p* = 0.88
	Spinal cord	D_max_ (cGy)	Manual	4261 ± 161	–
			FD	4227 ± 191	*p* = 0.18
			UIH	4251 ± 281	*p* = 0.86
	Brainstem	D_max_ (cGy)	Manual	5578 ± 803	–
			FD	5528 ± 717	*p* = 0.43
			UIH	5551 ± 706	*p* = 0.72
	Parotid_L	V_30_ (%)	Manual	57.78 ± 25.86	–
			FD	58.07 ± 26.73	*p* = 0.82
			UIH	57.37 ± 26.36	*p* = 0.77
		D_mean_ (cGy)	Manual	4365 ± 926	–
			FD	4381 ± 959	*p* = 0.65
			UIH	4307 ± 951	*p* = 0.33
	Parotid_R	V_30_ (%)	Manual	55.53 ± 23.68	–
			FD	55.16 ± 23.82	*p* = 0.78
			UIH	53.96 ± 24.88	*p* = 0.52
		D_mean_ (cGy)	Manual	4060 ± 508	–
			FD	4057 ± 538	*p* = 0.95
			UIH	3936 ± 608	*p* = 0.15
	Len_L	D_max_ (cGy)	Manual	592 ± 390	–
			FD	591 ± 387	*p* = 0.84
			UIH	588 ± 379	*p* = 0.68
	Len_R	D_max_ (cGy)	Manual	568 ± 385	–
			FD	529 ± 279	*p* = 0.34
			UIH	529 ± 281	*p* = 0.36
	Optic nerve_L	D_max_ (cGy)	Manual	3551 ± 2224	–
			FD	3493 ± 2276	*p* = 0.32
			UIH	3525 ± 2206	*p* = 0.69
	Optic nerve_R	D_max_ (cGy)	Manual	3612 ± 2115	–
			FD	3617 ± 2119	*p* = 0.94
			UIH	3784 ± 2113	*p* = 0.21
	Temporal lobe_L	D_max_ (cGy)	Manual	6376 ± 2126	–
			FD	6444 ± 2156	*p* = 0.05
			UIH	6397 ± 2125	*p* = 0.62
	Temporal lobe_R	D_max_ (cGy)	Manual	6430 ± 2143	–
			FD	6442 ± 2130	*p* = 0.76
			UIH	6390 ± 2123	*p* = 0.27
	Oral cavity	D_mean_ (cGy)	Manual	3933 ± 551	–
			FD	3928 ± 566	*p* = 0.73
			UIH	3895 ± 565	*p* = 0.12
	Larynx	D_mean_ (cGy)	Manual	3829 ± 153	–
			FD	3809 ± 169	*p* = 0.46
			UIH	3798 ± 195	*p* = 0.28

### The correlation between dosimetric differences and the geometric metrics

Table 4 shows the results of the correlation analysis between dosimetric differences and geometric metrics, there is no OARs shows strong correlation between its ∆Dose and all of four geometric metrics. The only significant correlation was found between the femoral head ΔD_mean_ and its geometric metric HD (*R* = 0.40, *p* = 0.01 for femoral head ΔD_mean_ vs. HD). Although the brainstem ΔD_max_ and its DICE was significantly correlated, this might be a statistical random error since the trend is contrary to our expectations. For detailed data, please refer to the Additional file 1: Supplement C, Figs. S2–S5).

**Table 4:** The correlation between dosimetric differences and the geometric metrics

Site	Structure	Dosimetric differences	Geometric metrics	Correlation analysis
Rectal	Bladder	ΔV_40_ (%)	DICE	*R* = -0.01, *p* = 0.95
			MDA	*R* = 0.17, *p* = 0.46
			Jaccard	*R* = -0.01, *p* = 0.95
			HD	*R* = 0.13, *p* = 0.60
		ΔD_mean_ (cGy)	DICE	*R* = -0.14, *p* = 0.55
			MDA	*R* = 0.06, *p* = 0.81
			Jaccard	*R* = -0.14, *p* = 0.55
			HD	*R* = 0.13, *p* = 0.59
	Femoral heads	ΔD_mean_ (cGy)	DICE	*R* = -0.16, *p* = 0.34
			MDA	*R* = 0.28, *p* = 0.08
			Jaccard	*R* = -0.16, *p* = 0.34
			HD	*R* = 0.40, *p* = 0.01
NPC	Eyes	ΔD_max_ (cGy)	DICE	*R* = 0.27, *p* = 0.09
			MDA	*R* = 0.16, *p* = 0.32
			Jaccard	*R* = 0.27, *p* = 0.09
			HD	*R* = 0.20, *p* = 0.22
	Spinal cord	ΔD_max_ (cGy)	DICE	*R* = 0.08, *p* = 0.74
			MDA	*R* = -0.21, *p* = 0.37
			Jaccard	*R* = 0.08, *p* = 0.74
			HD	*R* = -0.15, *p* = 0.51
	Brainstem	ΔD_max_ (cGy)	DICE	*R* = 0.68, *p* = 0.00
			MDA	*R* = -0.28, *p* = 0.24
			Jaccard	*R* = 0.68, *p* = 0.00
			HD	*R* = -0.12, *p* = 0.63
	Parotids	ΔV_30_ (%)	DICE	*R* = 0.02, *p* = 0.90
			MDA	*R* = 0.05, *p* = 0.76
			Jaccard	*R* = 0.02, *p* = 0.90
			HD	*R* = -0.15, *p* = 0.35
		ΔD_mean_ (cGy)	DICE	*R* = -0.16, *p* = 0.33
			MDA	*R* = 0.19, *p* = 0.23
			Jaccard	*R* = -0.16, *p* = 0.33
			HD	*R* = 0.06, *p* = 0.70
	Lens	ΔD_max_ (cGy)	DICE	*R* = 0.14, *p* = 0.39
			MDA	*R* = -0.14, *p* = 0.37
			Jaccard	*R* = 0.14, *p* = 0.39
			HD	*R* = -0.10, *p* = 0.56
	Optic nerves	ΔD_max_ (cGy)	DICE	*R* = -0.19, *p* = 0.23
			MDA	*R* = 0.06, *p* = 0.69
			Jaccard	*R* = -0.19, *p* = 0.23
			HD	*R* = -0.01, *p* = 0.96
	Temporal lobes	ΔD_max_ (cGy)	DICE	*R* = -0.16, *p* = 0.34
			MDA	*R* = 0.14, *p* = 0.38
			Jaccard	*R* = -0.16, *p* = 0.34
			HD	*R* = -0.04, *p* = 0.80
	Oral cavity	ΔD_mean_ (cGy)	DICE	*R* = -0.33, *p* = 0.16
			MDA	*R* = 0.30, *p* = 0.20
			Jaccard	*R* = -0.33, *p* = 0.16
			HD	*R* = 0.13, *p* = 0.59
	Larynx	ΔD_mean_ (cGy)	DICE	*R* = -0.24, *p* = 0.30
			MDA	*R* = 0.31, *p* = 0.18
			Jaccard	*R* = -0.24, *p* = 0.30
			HD	*R* = 0.24, *p* = 0.31

## Discussion

In this study, we assessed the dosimetric impacts of deep learning-based OARs auto-segmentation on nasopharyngeal and rectal cancers. Our results showed that deep learning-based OARs auto-segmentation had no significant impact on the PTV dose distribution or most OARs dose-volume metrics, while the correlation between the geometric metrics and OARs dosimetric differences was weak.

Two deep learning auto-segmentation systems were investigated. Both systems are under clinical testing in our institution. The clinical test for FD started in November 2018. Radiation oncologists can use this system for NPC and rectal OARs auto-segmentation in our institution. These auto-segmented contours were usually reviewed and modified by radiation oncologists before clinical approval. This process has been applied on more than 500 patients. For UIH, we started testing it in March 2019. Similar to the FD system, radiation oncologists are required to review auto-segmented contours before clinical approval. The preliminary feedback of these two systems can reduce radiation oncologists’ workload. More detailed data are being collected.

For quantitative geometric evaluation, both systems can provide similar performance for five OARs (eyes, parotids, lens, oral cavity and temporal lobes, *p* > 0.05 DICE). These results are similar to those reported in other researches [7, 11]. Although the differences for the spinal cord and brainstem were significant, the deviation value was small (approximately 0.04 in DICE and < 0.5 mm in MDA). Six OARs, including the bladder, femoral heads, spinal cord, brainstem, optic nerves and larynx, were significantly different between the two systems (*p* < 0.05, DICE). The reasons might be as follows.

FD can provide a better performance than UIH (*p* < 0.05 DICE) for the femoral heads, optic nerves, spinal cord, and larynx, which might be caused by the different OARs definitions between our clinical routine and UIH training data. For example, we did not include the femoral necks in femoral head segmentation. UIH included the femoral necks (Fig. 4c, red arrow). Additionally, OARs that do not have clear visible boundaries on CT images like temporal lobes can have large delineation variations (Additional file 1: Supplement D, Figs. S10). By retraining the auto-segmentation model on our institution data, these deviations might be eliminated.

The performance of the bladder for FD was worse than that for UIH (*p* < 0.05 DICE). This finding might have been caused by the algorithm difference between the two systems. Our system used a 2D U-Net network, which could have some outliers, as our previous study demonstrated [21, 22]. UIH used a two-phase algorithm, which was more robust according to region location.

In dosimetric analysis, no difference was found for the PTV target (*p* > 0.05). The most significant dose difference was rectal PTV D_2_ (Manual: 5349 ± 177 cGy, FD: 5384 ± 167 cGy, UIH: 5383 ± 160 cGy, *p* = 0.08). This study did not involve the auto-segmentation of target volume, all reoptimized plans used manually delineated PTV. The small dosimetric difference of PTV might be mainly caused by the experience, skills and operating habits of different dosimetrists. For OARs dose-volume metrics, the most significant dose difference was in the left temporal lobe D_max_ for Plan_FD vs. Plan_Manual (6376 ± 2126 cGy vs. 6444 ± 2156 cGy, *p* = 0.05). This finding might have been caused by the large variation in the delineation of the temporal lobes (Additional file 1: Supplement D, Fig. S10).

However, no significant dose-volume metrics difference was found for PTV and OARs. A plan dose distribution review remains necessary to fully investigate the dosimetric impact of an auto-segmentation system. The delineation could have a different impact on the final dose distribution. As we demonstrated in Fig. 4c, g, the femoral neck delineated by the UIH system was spared from 10% dose coverage. The low-dose isodose lines (10 Gy and 25 Gy) of Plan_UIH have different shapes compared to Plan_Manual and Plan_FD. In contrast, the difference between oral cavity delineation for UIH and manual delineation did not cause a significant dose distribution difference (Additional file 1: Supplement B, Fig. S1. C and G, red arrow).

This study showed that there was no clear monotonic relationship between the geometric metrics and dosimetric differences for most OARs. The only significant correlation was shown for the femoral head mean dose. There could be several reasons for this result. First, the difference between manual and automatic delineation might be too small to cause a dosimetric difference beyond the random noise dose levels. In other words, the performance of our two auto-segmentation systems was “good enough”. When the delineation difference is sufficiently large, such as with the femoral head definition, the correlation between geometric metrics and dosimetric difference can still be observed. Second, the interoperator difference or intraoperator difference during treatment planning could cause a larger difference than auto-segmentation. These interoperator differences were difficult to avoid by the manual planning process. By automatic planning, these subjective deviations can be decreased. To analyze the impact on routine clinical practice, we did not implement it.

In this study, we used manually delineated contours as references. This fact does not mean that manual delineation is “better” or more “accurate” than deep learning-based delineation. In our ongoing evaluation study, radiation oncologists preferred auto-segmented contours over manual delineation for the parotids, optic nerves, lens and eyes. This phenomenon was also observed in [11]. Manual delineation represents a clinically acceptable and approved contour quality, which also implies some clinical experience or the habits of local institutions. Therefore, using a commercial auto-segmentation system that is not trained on local data requires more investigation.

For segmentation evaluation, geometric evaluation is a straightforward method for auto-segmentation performance. Many studies using these indices have been published in recent years [17, 27–29]. Geometric metrics, such as DICE and MDA, are the critical indices for segmentation algorithm development. Using high-quality and consistent training or validation data, the algorithm performance can be quantified and compared. However, the clinical assessment of auto-segmentation can be much more complicated and should be based on clinical purposes. A small improvement in geometric metrics, for example, DICE increase of 0.05, could represent substantial progress in the algorithm. However, its clinical value is likely to improve only marginally. A more practical assessment procedure should mimic clinical practice as much as possible. This principle is also consistent with some task-based evaluation procedures proposed by other studies [30, 31].

The main limitation of this study was that it did not investigate interoperator variations. Using the auto-planning technique might reduce these variations, in turn increasing objectivity when plans are compared. These tasks were left for the future to complete.

## Conclusion

Deep learning-based OARs auto-segmentation for NPC and rectal cancer might not have a significant impact on PTV and OARs doses. Correlations between the auto-segmentation geometric metric and dosimetric difference were not observed for most OARs. A dosimetric evaluation is recommended for applying auto-segmentation systems in the clinic.

## Supplementary Information

Additional file 1. **Supplement A**. The details of the patient characteristics. **Supplement B**. Representative nasopharyngeal carcinoma examples of auto-segmentation and dose distribution. **Supplement C**. The correlation analysis between the geometric metrics and dosimetric differences. **Supplement D**. More rectal cancer and nasopharyngeal carcinoma examples of auto-segmentation and dose distribution.

## Data Availability

The datasets used and/or analyzed during the current study are available from the corresponding author on reasonable request.

## Abbreviations

OARs: Organs at risk
NPC: Nasopharyngeal carcinoma
sIMRT: Static intensity modulated radiotherapy
HD: Hausdorff distance
MDA: Mean distance to agreement
DICE: Dice similarity coefficient
HI: Homogeneity index
CI: Conformity index (CI)

## References

[1] Brodin NP, Kabarriti R, Garg MK (2018). Systematic review of normal tissue complication models relevant to standard fractionation radiation therapy of the head and neck region published after the QUANTEC reports. Int J Radiat Oncol Biol Phys.

[2] Liu Y, Lei Y, Wang Y (2019). MRI-based treatment planning for proton radiotherapy: dosimetric validation of a deep learning-based liver synthetic CT generation method. Phys Med Biol.

[3] Wang J, Qing G, Ou X (2019). The impact of target dosimetry on patients' locoregional recurrence in nasopharyngeal carcinoma: a propensity score-matched analysis. Radiother Oncol.

[4] Ma JL, Hennessey DB, Newell BP (2018). Radiotherapy-related complications presenting to a urology department: a more common problem than previously thought?. BJU Int.

[5] Kim N, Chang JS, Kim YB (2020). Atlas-based auto-segmentation for postoperative radiotherapy planning in endometrial and cervical cancers. Radiat Oncol.

[6] Cardenas CE, Yang J, Anderson BM (2019). Advances in auto-segmentation. Semin Radiat Oncol.

[7] Wong J, Fong A, McVicar N (2020). Comparing deep learning-based auto-segmentation of organs at risk and clinical target volumes to expert inter-observer variability in radiotherapy planning. Radiother Oncol.

[8] Ayyalusamy A, Vellaiyan S, Subramanian S (2019). Auto-segmentation of head and neck organs at risk in radiotherapy and its dependence on anatomic similarity. Radiat Oncol J.

[9] Elguindi S, Zelefsky MJ, Jiang J (2019). Deep learning-based auto-segmentation of targets and organs-at-risk for magnetic resonance imaging only planning of prostate radiotherapy. Phys Imaging Radiat Oncol.

[10] Savenije MHF, Maspero M, Sikkes GG (2020). Clinical implementation of MRI-based organs-at-risk auto-segmentation with convolutional networks for prostate radiotherapy. Radiat Oncol.

[11] van Dijk LV, Van den Bosch L, Aljabar P (2020). Improving automatic delineation for head and neck organs at risk by Deep Learning Contouring. Radiother Oncol.

[12] Vergalasova I, Cai J (2020). A modern review of the uncertainties in volumetric imaging of respiratory-induced target motion in lung radiotherapy. Med Phys.

[13] Sharp G, Fritscher KD, Pekar V (2014). Vision 20/20: perspectives on automated image segmentation for radiotherapy. Med Phys.

[14] Choi MS, Choi BS, Chung SY (2020). Clinical evaluation of atlas- and deep learning-based automatic segmentation of multiple organs and clinical target volumes for breast cancer. Radiother Oncol.

[15] Zabel WJ, Conway JL, Gladwish A, et al. Clinical Evaluation of Deep Learning and Atlas-Based Auto-Contouring of Bladder and Rectum for Prostate Radiation Therapy. Pract Radiat Oncol 2020. 10.1016/j.prro.2020.05.013 32599279

[16] Chen W, Li Y, Dyer BA (2020). Deep learning vs. atlas-based models for fast auto-segmentation of the masticatory muscles on head and neck CT images. Radiat Oncol.

[17] van der Veen J, Willems S, Deschuymer S (2019). Benefits of deep learning for delineation of organs at risk in head and neck cancer. Radiother Oncol.

[18] Ibragimov B, Xing L (2017). Segmentation of organs-at-risks in head and neck CT images using convolutional neural networks. Med Phys.

[19] Zhu W, Huang Y, Zeng L (2019). AnatomyNet: Deep learning for fast and fully automated whole-volume segmentation of head and neck anatomy. Med Phys.

[20] Kaderka R, Gillespie EF, Mundt RC (2019). Geometric and dosimetric evaluation of atlas based auto-segmentation of cardiac structures in breast cancer patients. Radiother Oncol.

[21] Wang J, Lu J, Qin G (2018). Technical note: a deep learning-based auto segmentation of rectal tumors in MR images. Med Phys.

[22] Xia X, Wang J, Li Y (2020). An artificial intelligence-based full-process solution for radiotherapy: a proof of concept study on rectal cancer. Front Oncol.

[23] Fan J, Wang J, Chen Z (2019). Automatic treatment planning based on three-dimensional dose distribution predicted from deep learning technique. Med Phys.

[24] Fan J, Xing L, Dong P (2020). Data-driven dose calculation algorithm based on deep U-Net. Phys Med Biol.

[25] Liu J, Zhang J, Shao Y, et al. Radiation therapy system. US Patent 9974980, 2018-5-22.

[26] Zhou J, Wang L, Ni C, et al. Systems and methods for generating radiation treatment plan. US Patent Application 16/109707, 2019-11-7.

[27] Taha AA, Hanbury A (2015). Metrics for evaluating 3D medical image segmentation: analysis, selection, and tool. BMC Med Imaging.

[28] Heimann T, van Ginneken B, Styner MA (2009). Comparison and evaluation of methods for liver segmentation from CT datasets. IEEE Trans Med Imaging.

[29] Kuperman VY, Figueiredo G (2020). Technical Note: New similarity index for radiotherapy and medical imaging. Med Phys.

[30] Barrett HH, Myers KJ, Hoeschen C (2015). Task-based measures of image quality and their relation to radiation dose and patient risk. Phys Med Biol.

[31] Conzelmann J, Schwarz FB, Hamm B (2020). Development of a method to create uniform phantoms for task-based assessment of CT image quality. J Appl Clin Med Phys.

