# Supplementary material for: The dosimetric impact of deep learning-based auto-segmentation of organs at risk on nasopharyngeal and rectal cancer
Source: Radiat Oncol. 2021 Jun 23;16:113. doi: 10.1186/s13014-021-01837-y (PMC8220801; doi:10.1186/s13014-021-01837-y)
Supplement: Supplementary file 1 — Additional file 1. Supplement A. The details of the patient characteristics. Supplement B. Representative nasopharyngeal carcinoma examples of auto-segmentation and dose distribution. Supplement C. The correlation analysis between the geometric metrics and dosimetric differences. Supplement D. More rectal cancer and nasopharyngeal carcinoma examples of auto-segmentation and dose distribution. [file 13014_2021_1837_MOESM1_ESM.docx]

**1. Supplement A**

**Table S1.** Patient characteristics.

| Site | NPC | Rectum |
| --- | --- | --- |
| Age | 57±15 | 56±13 |
| Gender |  |  |
| Male | 6 | 3 |
| Female | 4 | 7 |
| T-stage |  |  |
| T1-T2 | 3 | 2 |
| T3-T4 | 7 | 8 |
| N-stage |  |  |
| N0-N1 | 5 | 3 |
| N2-N3 | 5 | 7 |

**2. Supplement B**


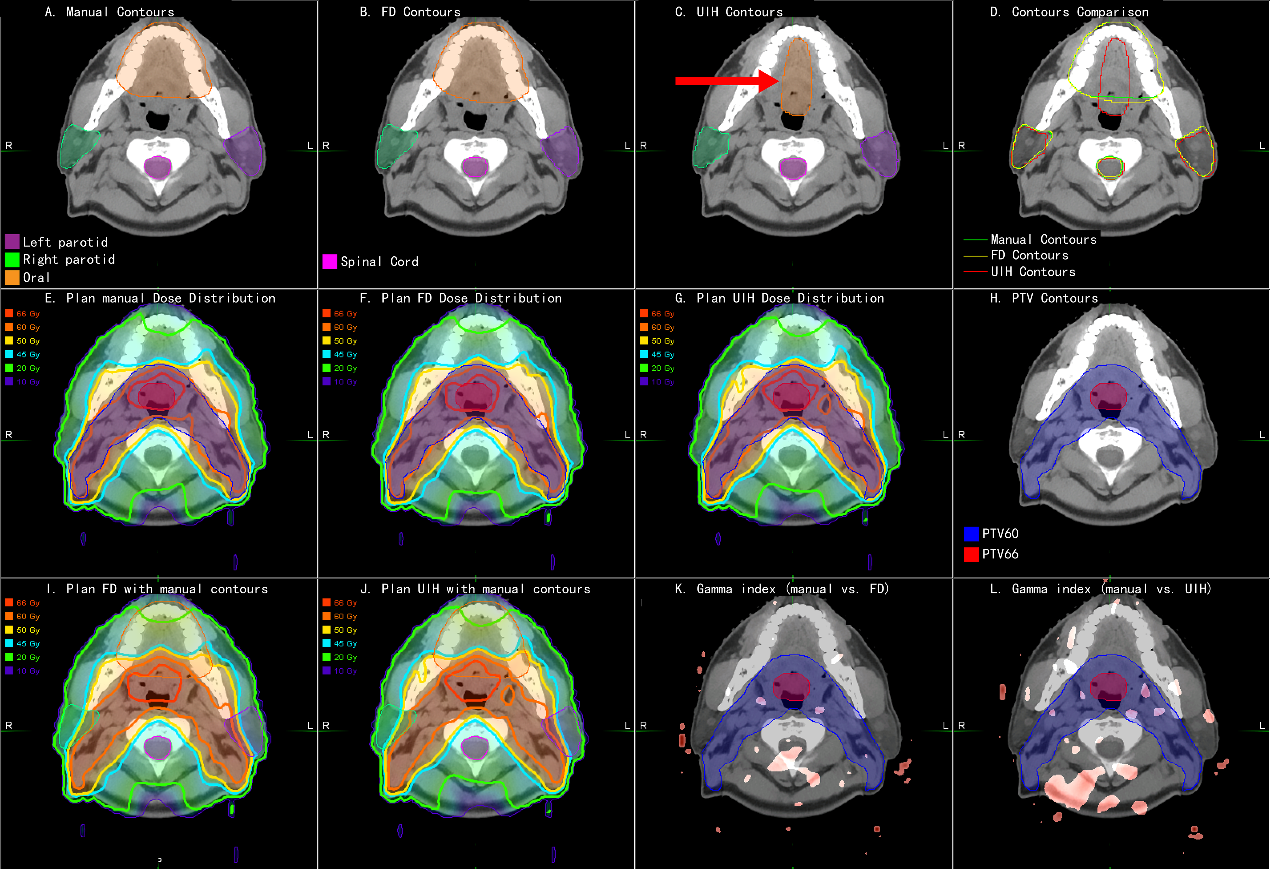


**Fig. S1** An example of nasopharyngeal carcinoma (NPC) patient. A. Manual OARs; B. FD OARs; C. UIH OARs; D. Contour comparison; E. Plan_Manual dose distribution; F. Plan_FD dose distribution; G. Plan_UIH dose distribution; H. PTV contour; I. Plan_FD with manual OARs; J. Plan_UIH with manual OARs; K. 3D gamma analysis of Plan_FD; red color represents gamma index > 1; L. 3D gamma analysis of Plan_UIH, red color represents gamma index > 1.

**Table S2.** Summary of the OARs dosimetry parameters of the original treatment plans (Plan_Manual) on orginal OARs (OAR_Manual) and two deep learning-based segmentations (OAR_FD and OAR_UIH). All the values are reported as the mean ± standard deviation.

| **Site** | **Structure** | **Dosimetric metrics** | **OARs sets** | **Value** | **Comparison (paired t test)** |
| --- | --- | --- | --- | --- | --- |
| Rectal | Bladder | V_40_ (%) | Manual | 43.45 ± 26.96 | / |
|  |  |  | FD | 42.85 ± 24.93 | *p* = 0.83 |
|  |  |  | UIH | 42.55 ± 25.74 | *p* = 0.54 |
|  |  | D_mean_ (cGy) | Manual | 3476 ± 1281 | / |
|  |  |  | FD | 3451 ± 1216 | *p* = 0.70 |
|  |  |  | UIH | 3401 ± 1250 | *p* = 0.11 |
|  | Femoral head_L | D_mean_ (cGy) | Manual | 2199 ± 773 | / |
|  |  |  | FD | 2198 ± 714 | *p* = 0.99 |
|  |  |  | UIH | 2134 ± 859 | *p* = 0.59 |
|  | Femoral head_R | D_mean_ (cGy) | Manual | 2130 ± 755 | / |
|  |  |  | FD | 2084 ± 751 | *p* = 0.53 |
|  |  |  | UIH | 2125 ± 820 | *p* = 0.95 |
| NPC | Eye_L | D_max_ (cGy) | Manual | 2199 ± 1175 | / |
|  |  |  | FD | 2147 ± 1244 | *p* = 0.62 |
|  |  |  | UIH | 2151 ± 1163 | *p* = 0.71 |
|  | Eye_R | D_max_ (cGy) | Manual | 2441 ± 1892 | / |
|  |  |  | FD | 2461 ± 1845 | *p* = 0.89 |
|  |  |  | UIH | 2379 ± 1873 | *p* = 0.74 |
|  | Spinal cord | D_max_ (cGy) | Manual | 4261 ± 161 | / |
|  |  |  | FD | 4225 ± 206 | *p* = 0.25 |
|  |  |  | UIH | 4246 ± 321 | *p* = 0.81 |
|  | Brainstem | D_max_ (cGy) | Manual | 5578 ± 803 | / |
|  |  |  | FD | 5530 ± 667 | *p* = 0.51 |
|  |  |  | UIH | 5523 ± 669 | *p* = 0.57 |
|  | Parotid_L | V_30_ (%) | Manual | 57.78 ± 25.86 | / |
|  |  |  | FD | 56.89 ± 32.01 | *p* = 0.20 |
|  |  |  | UIH | 56.99 ± 27.01 | *p* = 0.33 |
|  |  | D_mean_ (cGy) | Manual | 4365 ± 926 | / |
|  |  |  | FD | 4241 ± 1027 | *p* = 0.18 |
|  |  |  | UIH | 4308 ± 1024 | *p* = 0.59 |
|  | Parotid_R | V_30_ (%) | Manual | 55.53 ± 23.68 | / |
|  |  |  | FD | 55.38 ± 33.21 | *p* = 0.11 |
|  |  |  | UIH | 54.67 ± 25.61 | *p* = 0.25 |
|  |  | D_mean_ (cGy) | Manual | 4060 ± 508 | / |
|  |  |  | FD | 4015 ± 513 | *p* = 0.70 |
|  |  |  | UIH | 3848 ± 633 | *p* = 0.17 |
|  | Len_L | D_max_ (cGy) | Manual | 592 ± 390 | / |
|  |  |  | FD | 604 ± 388 | *p* = 0.20 |
|  |  |  | UIH | 554 ± 371 | *p* = 0.08 |
|  | Len_R | D_max_ (cGy) | Manual | 568 ± 385 | / |
|  |  |  | FD | 525 ± 256 | *p* = 0.39 |
|  |  |  | UIH | 510 ± 295 | *p* = 0.18 |
|  | Optic nerve_L | D_max_ (cGy) | Manual | 3551 ± 2224 | / |
|  |  |  | FD | 3460 ± 2253 | *p* = 0.44 |
|  |  |  | UIH | 3622 ± 2337 | *p* = 0.46 |
|  | Optic nerve_R | D_max_ (cGy) | Manual | 3612 ± 2115 | / |
|  |  |  | FD | 3600 ± 2154 | *p* = 0.95 |
|  |  |  | UIH | 3877 ± 1960 | *p* = 0.22 |
|  | Temporal lobe_L | D_max_ (cGy) | Manual | 6376 ± 2126 | / |
|  |  |  | FD | 6579 ± 2178 | *p* = 0.10 |
|  |  |  | UIH | 6356 ± 2289 | *p* = 0.86 |
|  | Temporal lobe_R | D_max_ (cGy) | Manual | 6430 ± 2143 | / |
|  |  |  | FD | 6320 ± 2070 | *p* = 0.37 |
|  |  |  | UIH | 6454 ± 2193 | *p* = 0.86 |
|  | Oral cavity | D_mean_ (cGy) | Manual | 3933 ± 551 | / |
|  |  |  | FD | 3922 ± 552 | *p* = 0.83 |
|  |  |  | UIH | 3799 ± 561 | *p* = 0.06 |
|  | Larynx | D_mean_ (cGy) | Manual | 3829 ± 153 | / |
|  |  |  | FD | 3884 ± 215 | *p* = 0.43 |
|  |  |  | UIH | 3801 ± 254 | *p* = 0.66 |

**3. Supplement C**


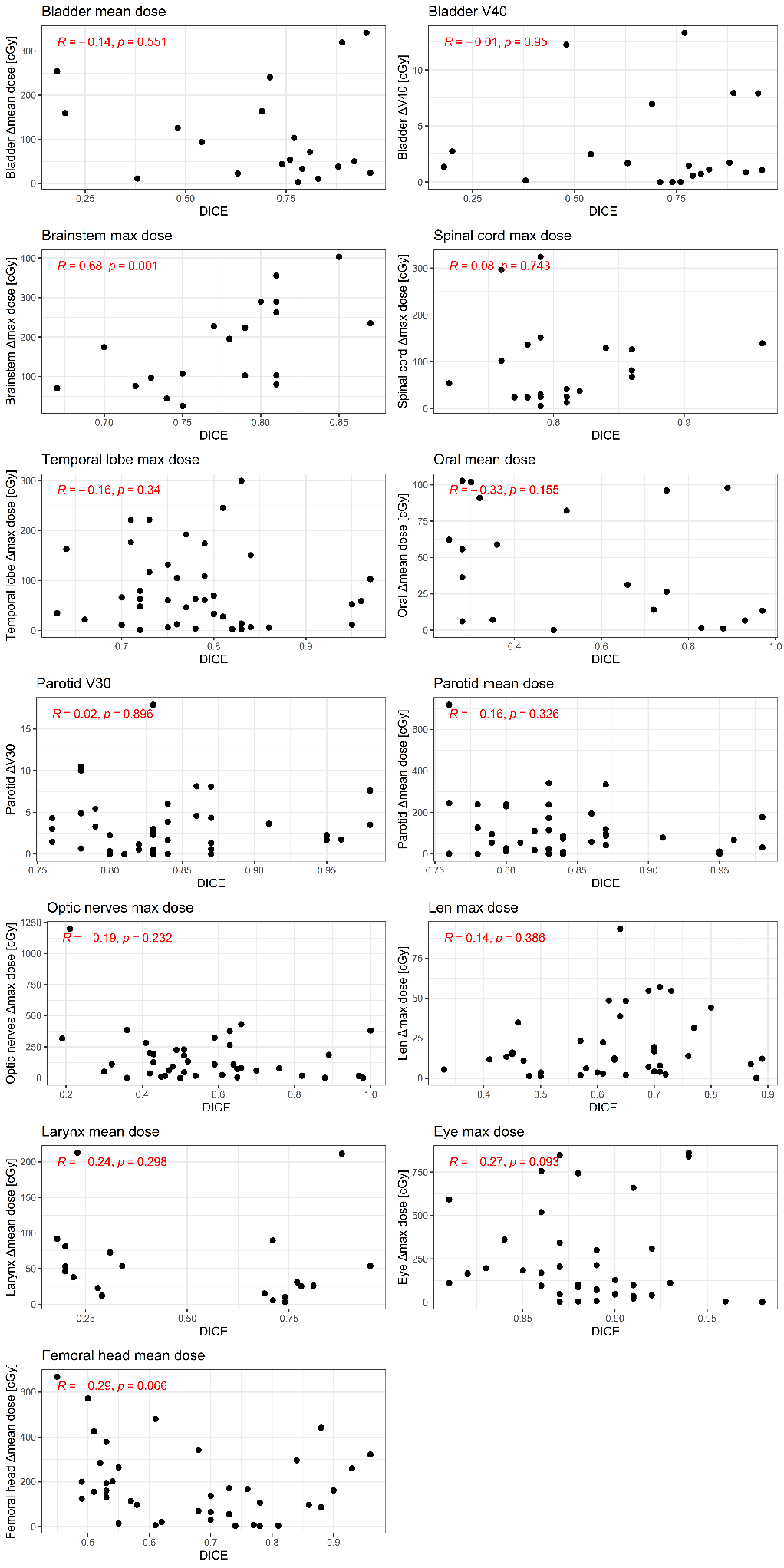


**Fig. S2** The correlation between DICE and dose metric deviation





**Fig. S3** The correlation between Hausdorff distance and dose metric deviation





**Fig. S4** The correlation between JACCARD and dose metric deviation





**Fig. S5** The correlation between mean distance to agreement and dose metric deviation

**4. Supplement D**


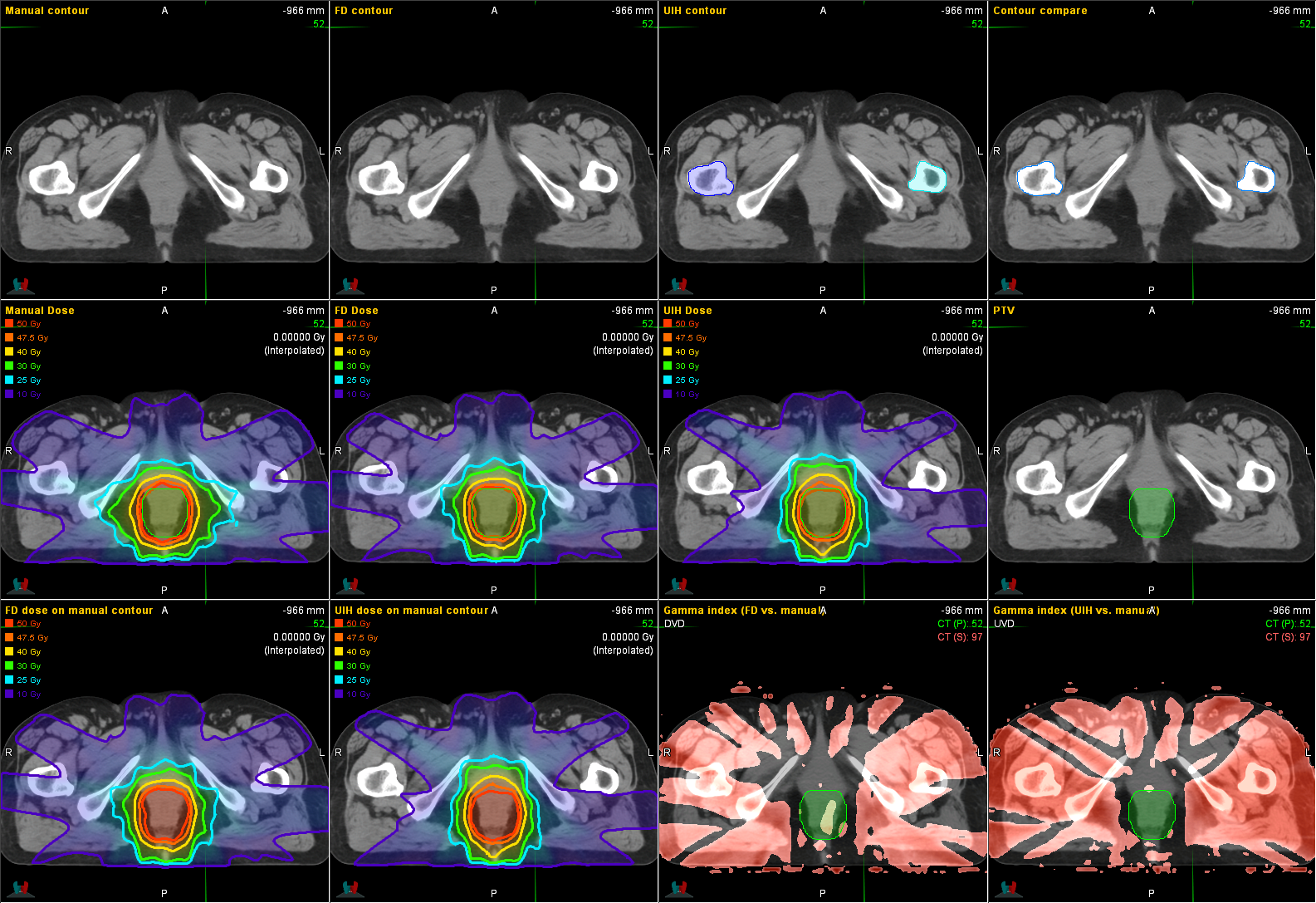


**Fig. S6** Rectal cancer patient example


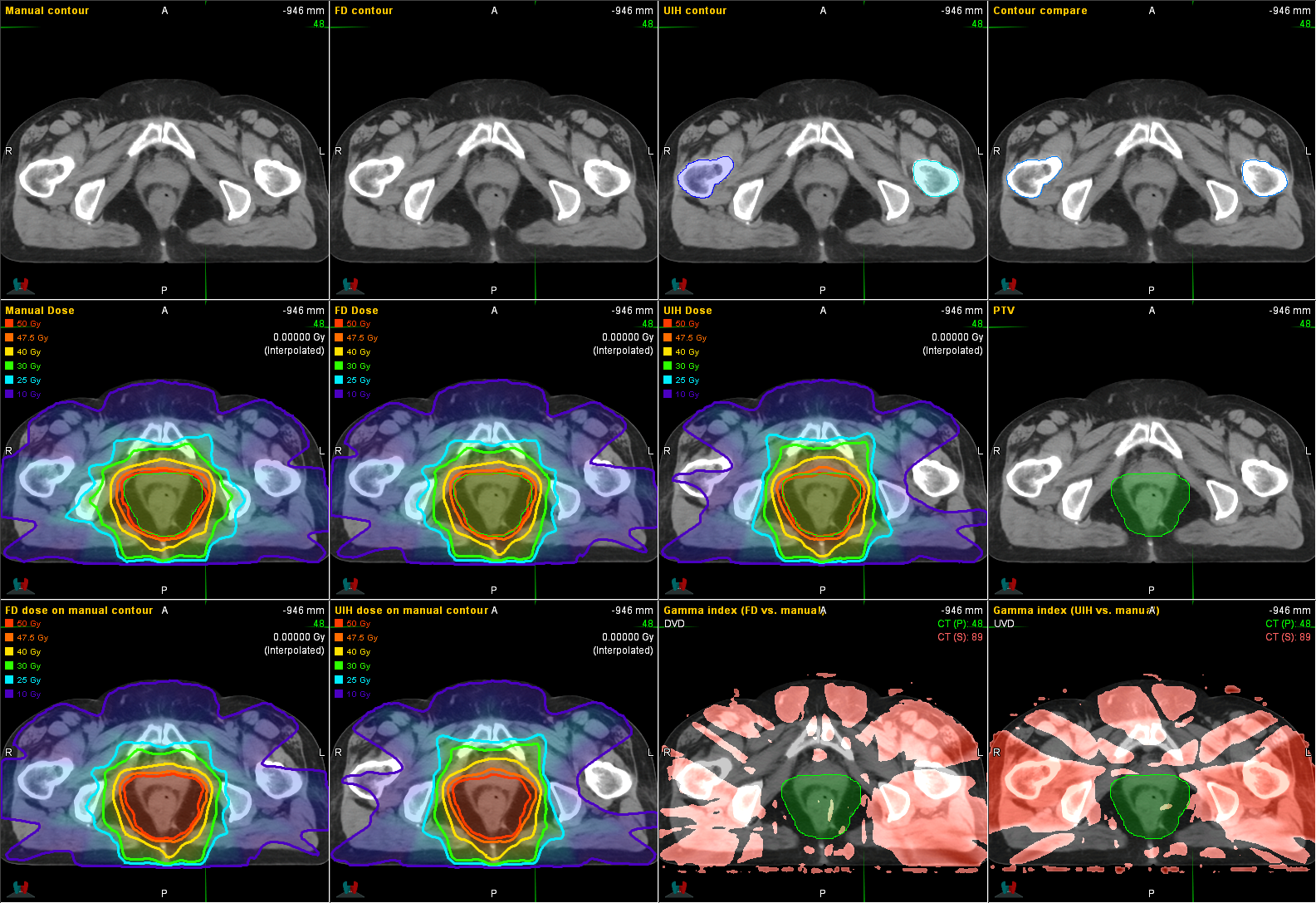


**Fig. S7** Rectal cancer patient example


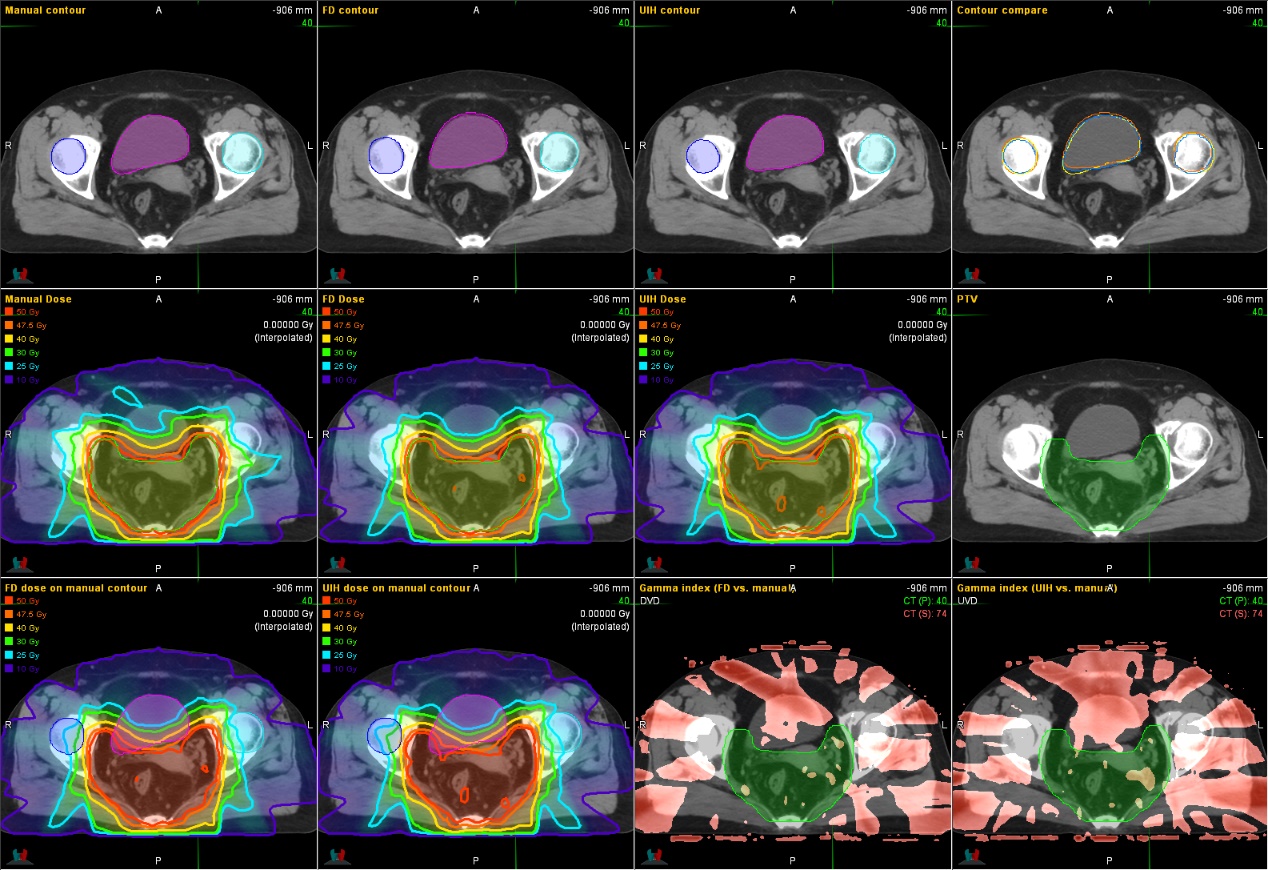


**Fig. S8** Rectal cancer patient example


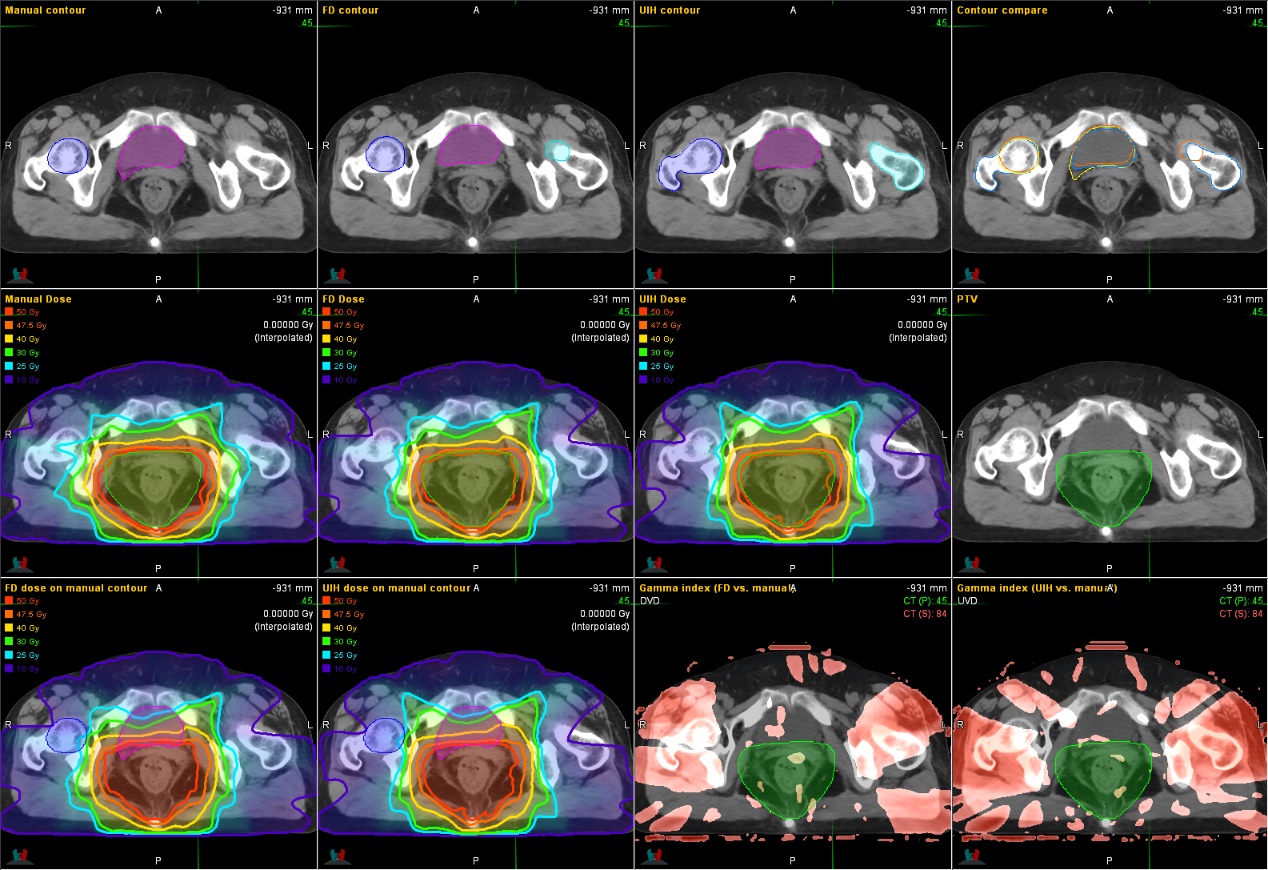


**Fig. S9** Rectal cancer patient example


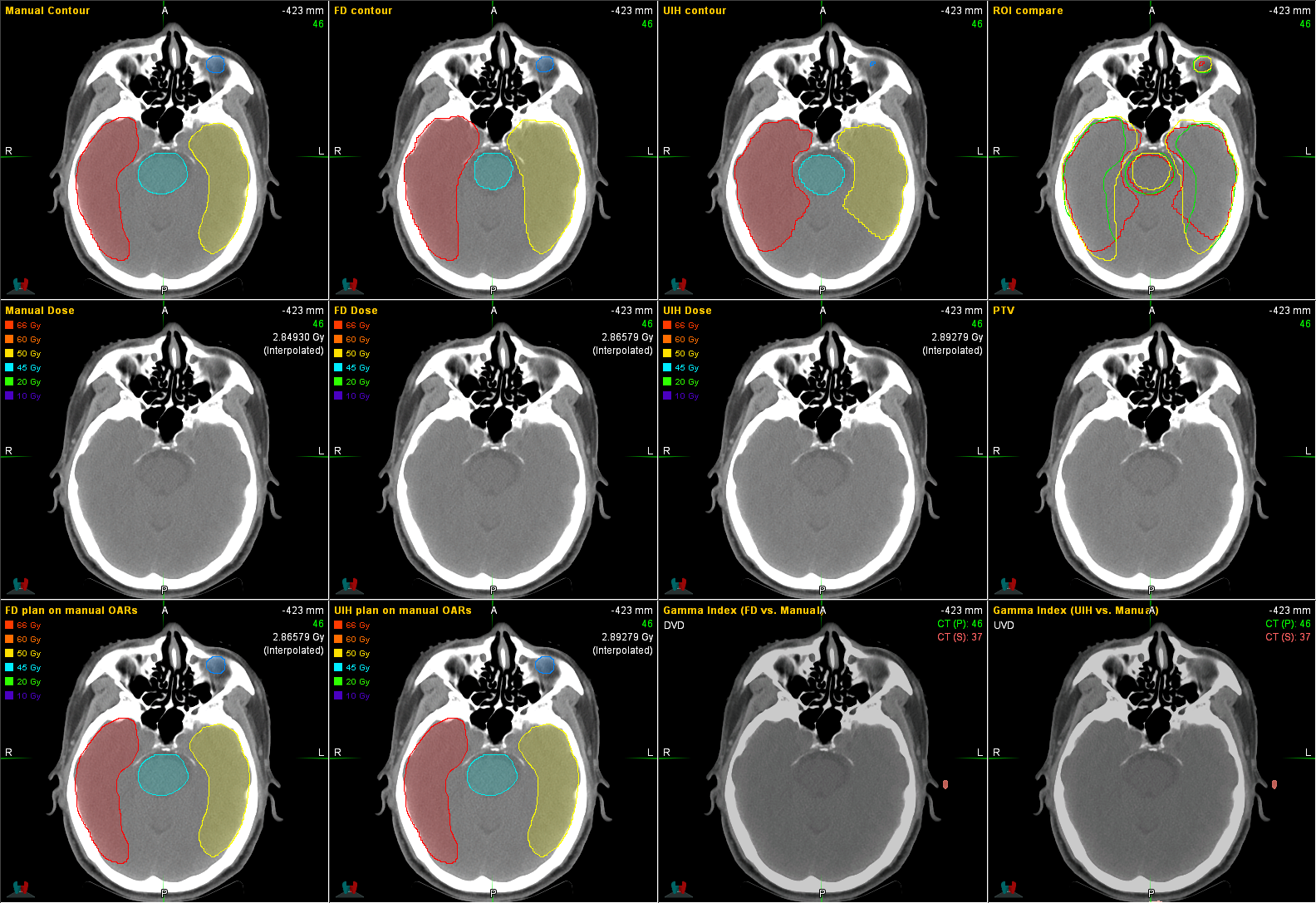


**Fig. S10** NPC patient example


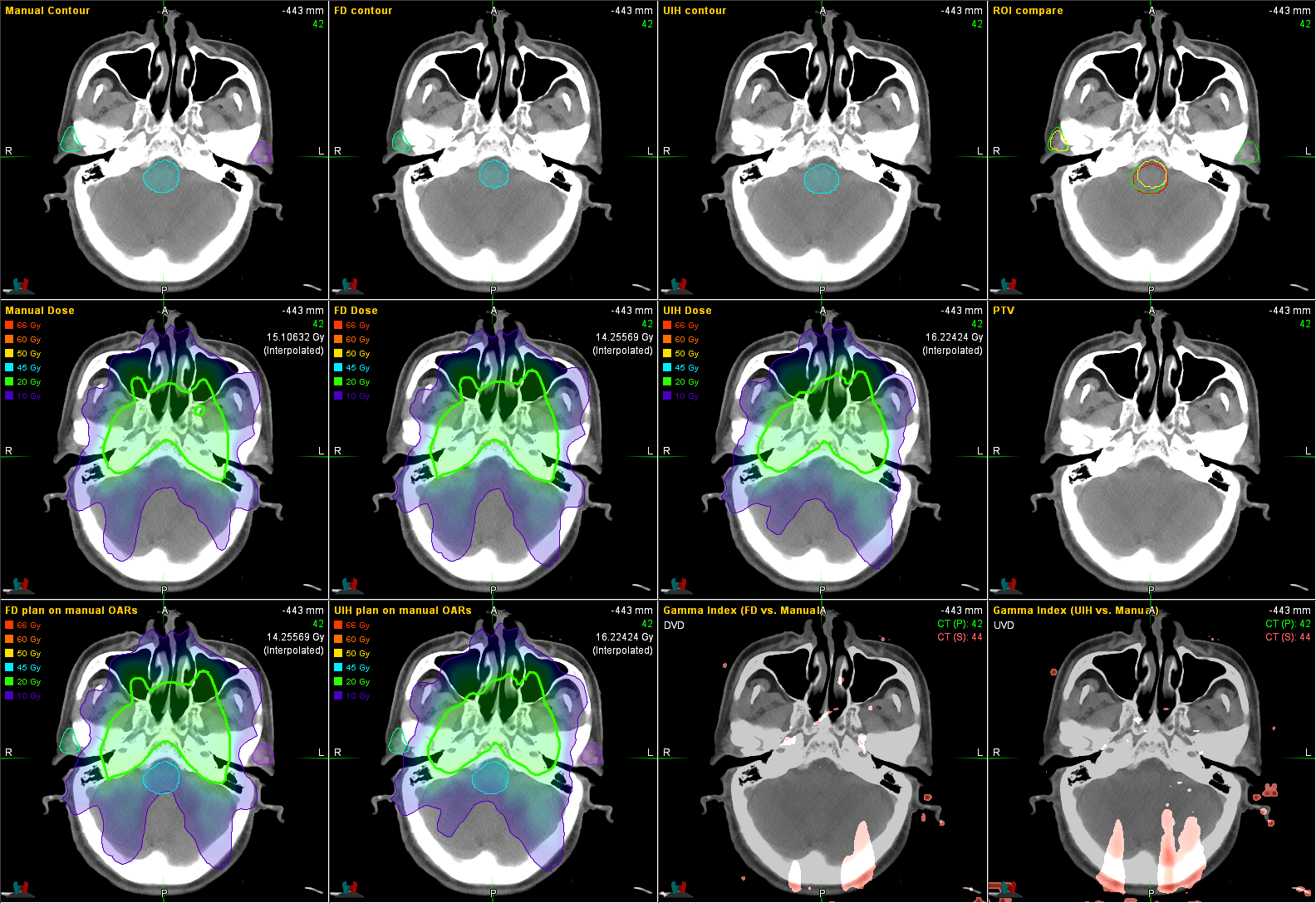


**Fig. S11** NPC patient example


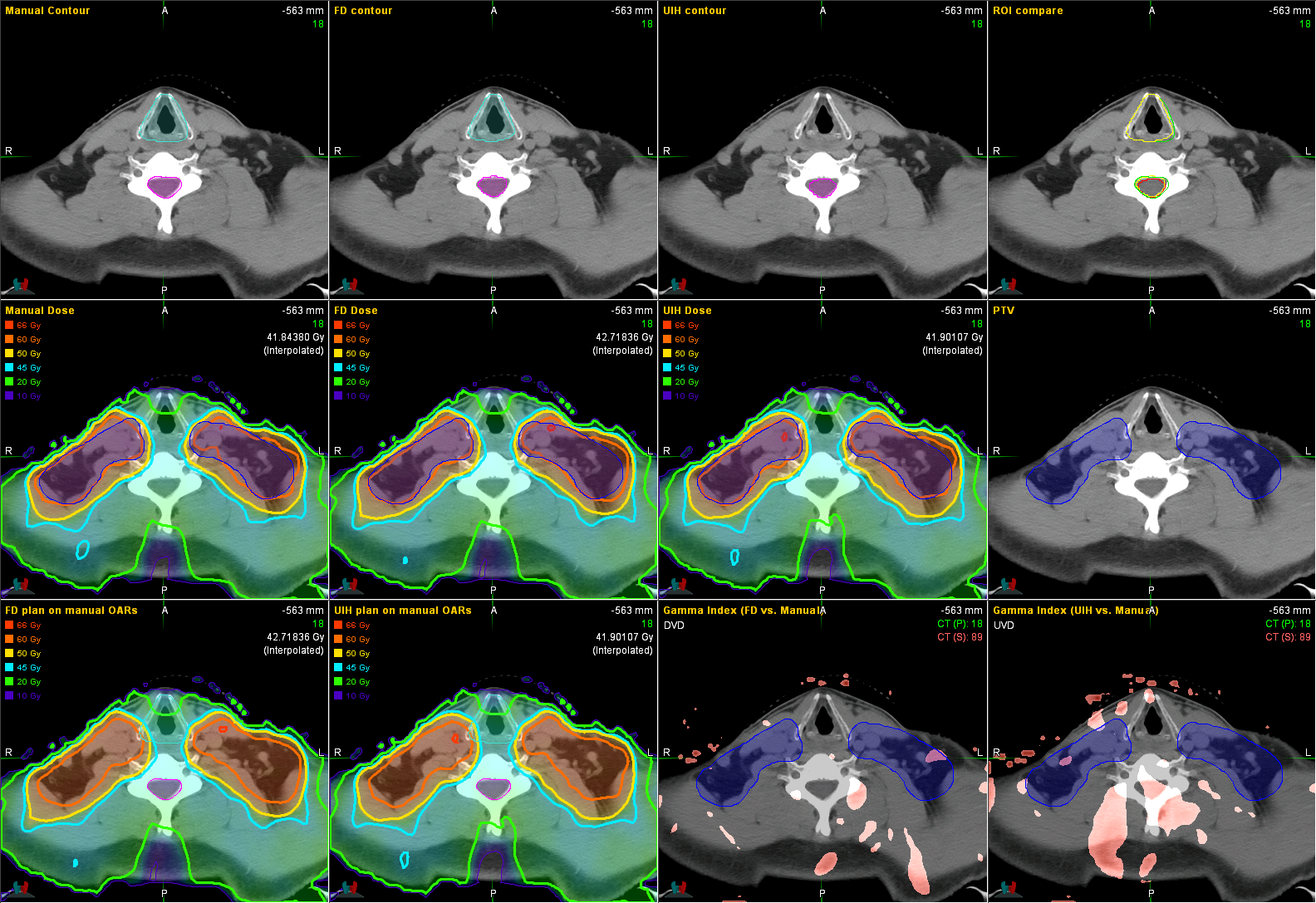


**Fig. S12** NPC patient example
